# Comparison of Soft Tissue Cephalometric Norms between Turkish and European-American Adults

**DOI:** 10.1155/2013/806203

**Published:** 2013-03-07

**Authors:** Ahmet Arif Celebi, Enes Tan, Ibrahim Erhan Gelgor, Tugba Colak, Erdem Ayyildiz

**Affiliations:** Department of Orthodontics, Faculty of Dentistry, Kirikkale University, Kirikkale, 71100 Merkez, Turkey

## Abstract

One of the most important components of orthodontic diagnosis and treatment planning is the evaluation of the patient's soft tissue profile. The main purpose of this study was to develop soft-tissue cephalometric standards for Turkish men and women and compare them with the cephalometric standards of normal European-American white people. The sample included 96 Turkish adults (48 women, 48 men), aged 20 to 27 years. Turkish subjects have increased facial convexity associated with retruded mandible, more obtuse lower face-throat angle, increased nasolabial angle and upper lip protrusion, deeper mentolabial sulcus, and smaller interlabial gap compared with European-American white people. It is appropriate to consider these differences during routine diagnosis and treatment planning of a Turkish patient or an American patient of European ancestry. Turkish males reveal more obtuse mandibular prognathism and upper lip protrusion, and smaller nasolabial angle than females.

## 1. Introduction

When Broadbent introduced his cephalometer in 1931, a new period began in orthodontics. The aim of cephalometric analyses is to determine the relationships of the dentofacial complex. Cephalograms can also help the orthodontist determine the changes associated with growth and orthodontic treatment [1]. Several cephalometric analyses have been applied to defined samples of untreated individual to derive cephalometric “norms” or “standards” for a given population, usually defined in terms of age and ethnic origin [2, 3]. All of them presented average measurements of skeletal and/or dental patterns with their ranges in a sample. Whereas some investigators have noted the importance of the soft tissue in the determination of facial aesthetics on the basis that soft tissue behaves independently from the underlying skeleton because of the individual differences in soft tissue thickness [4–7].

The successful treatment planning for patients who require orthognathic surgery should include both hard and soft tissue cephalometric analysis [8]. Various cephalometric analyses for orthognathic surgery have been designed [5, 6, 9–12]. Legan and Burstone soft tissue analysis is one of the most common analysis systems used for orthognathic surgery [6, 13, 14]. However, these cephalometric norms were specific to 1 ethnic group—white subjects of European-American ancestry—and might not apply to other ethnic groups. Cephalometric norms for different ethnic and racial groups have previously been established in many studies [15–19]. Therefore, it is important to develop norms of various populations with a standard method.

Turkey is an Eurasian country located in Western Asia (mostly in the Anatolian peninsula) and in Southeastern Europe (East Thracian). Turkish population has genes from Asiatic Turks, Kurds, the Balkans, Caucasus, Middle East, Iran as well as from ancient Romans, Byzantines, and Arabs. Several studies aimed to determine the Turkish population's ideal norms. Erbay et al. [17] investigated cephalometrically the horizontal lip position of Anatolian Turkish adults using the soft tissue analyses of Steiner, Ricketts, Burstone, Sushner, and Holdaway. In another study, Erbay and Caniklioglu [18] evaluated the soft tissue profile to determine orthodontists' perceptions of Anatolian Turkish adults' beauty. Uysal et al. [20] determined cephalometrically the soft tissue norms of Anatolian Turkish adults using the soft tissue analyses of Arnett et al. [11]. Gelgor et al. used a parental data to evaluate soft tissues in an Anatolian Turkish population according to Holdaway soft tissue norms [21]. Uysal et al. [22], comparing Turkish and European-American adults, reported some significant differences in their soft tissue parameters.

Among the several numeric facial analyses currently used, the analysis proposed by Legan and Burstone is important, because it has been broadly used by orthodontists and maxillofacial surgeons in diagnosis and treatment planning. However, the measurements in this analysis were based on European-American samples and might not apply to Turkish patients, demonstrating the need for specific studies for this ethnic group. The aims of this study were (1) to determine norms for Legan and Burstone analysis from lateral cephalograms of Turkish adults and (2) to identify possible gender differences between males and females.

## 2. Materials and Methods

The study group included 96 (48 males, mean age: 22,6 and 48 female, mean age: 21,4) Turkish adults who were chosen from 2825 patients and who visited the Department of Orthodontics at Kirikkale Dental Faculty from 2004 to 2011. The following criteria were used for selection of the sample:

1. angle Class I occlusal relationship with normal overbite and overjet;
2. well-aligned upper and lower dental arches with minimal dental crowding;
3. normal growth and development pattern;
4. no history of previous orthodontic treatment, prosthodontic treatment or facial surgery.

Lateral cephalometric radiographs were taken according to the following criteria: (1) natural head position with teeth in centric occlusion, (2) the lips in rest position, and (3) the lateral cephalometric radiograph was taken at a standard source-to-cassette holder distance of 130 mm (magnification, 1.09) with a Planmeca Cephalometer (PM 2002 EC Proline, Helsinki, Finland). The subject was asked to look into the reflection of his/her own eyes in the mirror to obtain a natural head position.

**Figure 1:**
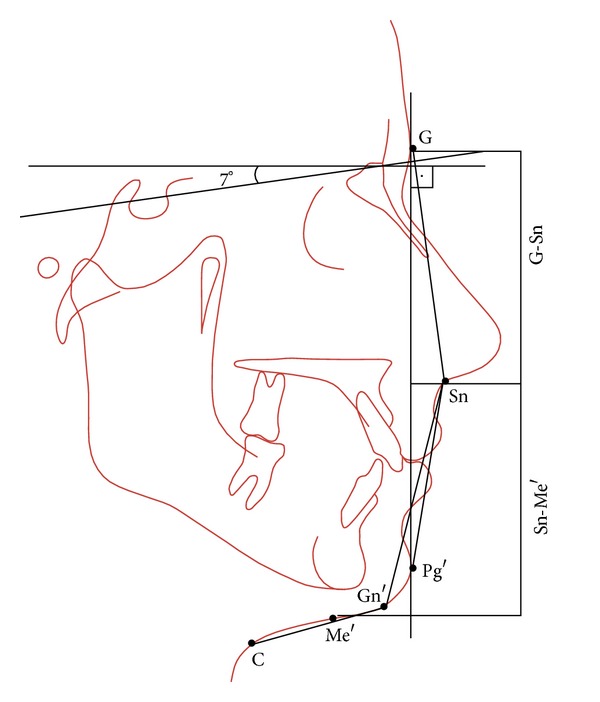
Legan-Burstone soft tissue analysis: facial forms. Horizontal reference plane (HP), constructed by drawing a line through nasion (N) 7 degrees up from the sella-nasion line. Facial convexity angle (G-Sn-Pg′); maxillary prognathism (G vertical-Sn); mandibular prognathism (G vertical-Pg′); vertical height ratio (G-Sn/Sn-Me′); lower face-throat angle (Sn-Gn′-C); lower vertical height-depth ratio (Sn-Gn′/C-Gn′).

**Figure 2:**
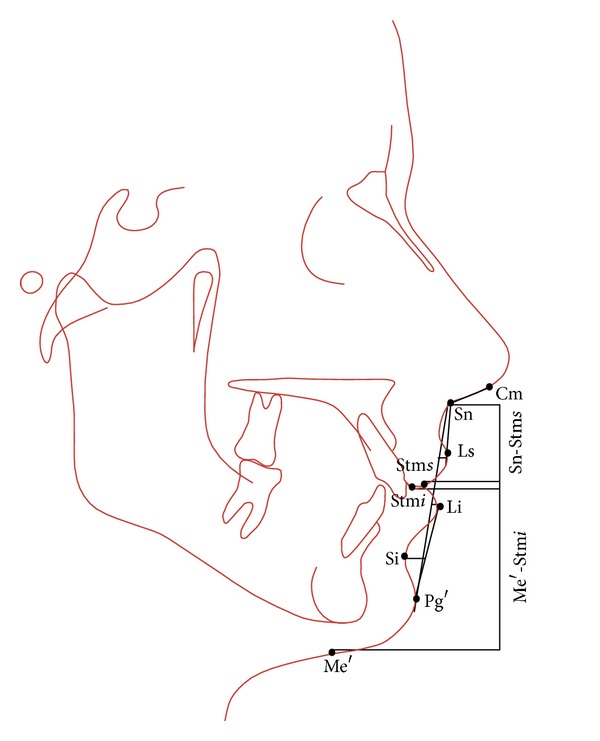
Legan-Burstone soft tissue analysis: lip position. Nasolabial angle (Cm-Sn-Ls); upper lip protrusion (Ls to Sn-Pg′); lower lip protrusion (Li to Sn-Pg′); mentolabial sulcus (Si to Li-Pg′); vertical lip-chin ratio (Sn-Stms/Stmi-Me′); maxillary incisor exposure (Stms-UI); interlabial gap (Stms-Stmi).

Landmarks and angular and linear measurements of the soft-tissue analysis of Legan and Burstone are shown in Figures 1 and 2.

All cephalometric parameters were measured on standardized lateral cephalometric radiographs by A.C. to test the reliability of the measurements, 20 randomly selected cephalograms were retraced 2 weeks later by the same orthodontist; all measurements were remeasured, and the reliabilities of the parameters were examined with analysis of variance index of reliability. The calculated reliabilities ranged from 89% to 98% and were statistically significant (*P* < 0.001). There were no significant differences between the mean values of each parameter.

All statistical analyses were performed using the Statistical Package for Social Sciences (Windows, version 13.0; SPSS Inc., Chicago, IL, USA). Descriptive statistics (mean, standard deviations) were assessed for each measurement in both sexes separately. An independent Student's *t*-test was used to test the gender differences and to compare the mean values of Turkish males and females with European-American's mean values originally obtained by Legan and Burstone analysis [6] at 5% level (*P* ≤ 0.05).

## 3. Results

The results showed descriptive statistics of the soft tissue cephalometric measurements for Turkish male and female subjects and compared them to European-American norms (Table 1). The Turkish norms had the following statistically significant differences: larger facial convexity angle (*P* = 0.035), lower face-throat angle (*P* = 0.004), nasolabial angle (*P* = 0.001), and upper lip protrusion (*P* = 0.005), more retruded pogonion (*P* = 0.043), deeper mentolabial sulcus (*P* = 0.001), and smaller interlabial gap (*P* = 0.046).

Table 2 illustrates the gender differences of soft tissue variables; no statistically significant differences are noticed except for the 3 variables. Turkish males revealed more obtuse mandibular prognathism (*P* = 0.018) and upper lip protrusion (*P* = 0.041), and smaller nasolabial angle (*P* = 0.027) than females.

## 4. Discussion

The nature of the soft tissue profile is affected by many factors, including ethnicity. For this reason, facial characteristics have been studied in various ethnic groups [14, 19, 23–26]. In our daily practice, various methods are used to evaluate cephalometric radiographs for orthognathic surgery. The advantage of such analyses is that they provide the ability to make objective evaluation of important structures and relationships [12]. In recent years the number of cephalometric studies has been increased for Turkish population. However, the applicability of the norms described in these analyses to Turkish people is controversial. Several attempts have been made to evaluate the soft tissues of the Turkish population [17, 18, 22, 27, 28].

The current study developed and compared cephalometric measurements of soft tissue facial profile of a sample of Turkish adults to European-American's norms using Legan and Burstone analysis.

The sample was limited to young adults with a mean age of 22 years because the vast majority of orthodontic patients are young adults [24, 29, 30].

Both Turkish males and females had a significant increase in facial convexity than the European-Americans as indicated by statistically significant larger facial convexity angle. The finding that the soft tissue facial angle is convex supports another previous study finding of similar convexity in soft tissue profile [22]. The angle of facial convexity in the Turkish population was smaller in males than in females. This indicates that males have relatively straighter facial profiles than females. However, the difference was not statistically significant. 

Maxillar prognathism did not show significant differences between the races. However, smaller values were recorded for the mandibular prognathism measurement in Turkish subjects compared to the European-American samples. Mandibular retrusion may be the reason for increased soft tissue convexity for Turkish sample.

The lower face-throat angle was more obtuse compared with European-American samples. However Al-Gunaid et al. [25] found that lower face-throat angle is not different between the races. An appreciation of this angle is critical in planning treatment to correct anteroposterior facial dysphasia. An obtuse lower face-throat angle should warn the clinician not to use procedures that reduce the prominence of the chin [6]. 

No statistically significant difference existed for the vertical ratio and lower vertical height-depth ratio between the races. Similar results were reported by Uysal et al. [22].

Turkish males and females had statistically significant larger nasolabial angle than those in European-American samples. Legan and Burstone [6] indicated that in surgical procedures this angle should be in the range of 102 ± 8 degrees. Turkish adult norms were near the upper border of the range and showed gender differences. The findings for nasolabial angle in the current investigation were distinctly different from those of Uysal et al. [20, 22].

Sex differences in upper lip protrusion Turkish subjects were statistically significant. It was obvious that the upper lips were more protrusive in the males. Similar results were reported by Uysal et al. [22]. Erbay et al. [17] determined lips protrusion in Anatolian Turkish adults using the esthetic plane analysis. According to the findings of Erbay et al. [17], upper and lower lips position is dentally and skeletally normal. Anatolian Turkish subjects were not statistically significant.

Turkish subjects had significantly more protruded upper lip positions than European-Americans. This finding was compatible with Uysal et al. [22]. No statistically significant difference existed for lower lip protrusion between the races and genders.

The mentolabial sulcus depth was significantly greater in Turkish adults than in the European-Americans; perhaps this might be attributed to mandibular retrusion.

The interlabial gap in Turkish subjects was significantly shorter compared with European-Americans which might be due to the difference in upper lip thickness.

When other ethnic groups were compared with European-Americans using Legan and Burstone analysis, significant differences were seen (Table 3). Chinese subjects had less convex faces, retrognathic chin, acute nasolabial angle, and more protrusive lips in comparison with European-Americans [14]. Japanese subjects had a retrognathic maxilla, retruded chin with less deep inferior sulcus, obtuse nasolabial angle, and more protrusive lips compared with European-Americans [24]. North Indian subjects had convex profile, more obtuse lower face-throat angle, protrusive lips, acute nasolabial angle, deep mentolabial sulcus, and shorter interlabial gap than in European-Americans [30]. Saudis have a more convex profile and reduced lower vertical height depth ratio values, shorter neck distance, and more reduced chin than European-Americans. In a study on a Yemeni population, soft tissue analyses showed a more convex facial form, a more retruded mandible, obtuse lower face throat angle, deep mentolabial sulcus, shorter interlabial gap, and increased incisor exposure compared with European-Americans.

In conclusion, the present study has produced normative cephalometric data for a Turkish population that will aid in diagnosis and treatment planning. In comparison with an European-American sample, Turkish subjects have increased facial convexity associated with retruded mandible, more obtuse lower face-throat angle, increased nasolabial angle and upper lip protrusion, more deep mentolabial sulcus, and smaller interlabial gap. 

## Figures and Tables

**Table 1:** Comparison of soft tissue cephalometric values of Turkish and European-American using Legan and Burstone analysis.

Variable	Turkish		European-American		*P* value
	Mean	SD	Mean	SD	
Facial form					
Facial convexity angle (°)	14,15	4,65	12	4	0.035*
Maxillary prognathism (mm)	5,5	3,85	6	3	0.724
Mandibular prognathism (mm)	−2.7	7,25	0	4	0.043*
Vertical height ratio	1,05	0,1	1	—	0.091
Lower face-throat angle (°)	105,65	8	100	7	0.004**
Lower vertical height-depth ratio	1,3	0,9	1,2	—	0.458
Lip position					
Nasolabial angle (°)	107,05	8,45	102	8	0.001***
Upper lip protrusion (mm)	3,35	1,9	3	1	0.005**
Lower lip protrusion (mm)	2,25	1,75	2	1	0.453
Mentolabial sulcus (mm)	−5.65	1,6	4	2	0.001***
Vertical lip-chin ratio	0,48	0.075	0,5	—	0.876
Maxillary incisor exposure (mm)	2,95	1,85	2	2	0.054
Interlabial gap (mm)	1,1	1,55	2	2	0.046*

*P* ≥ 0.05–nonsignificant (NS), *P ≤ 0.05, **P ≤ 0.01, ***P ≤ 0.001.

**Table 2:** Comparison of soft tissue cephalometric values of Turkish males and females using Legan and Burstone analysis.

Variable	Males		Females		*P* value
	Mean	SD	Mean	SD	
Facial form					
Facial convexity angle (°)	13,6	4,2	14,7	5,1	0.104
Maxillary prognathism (mm)	5,2	3,7	5,8	4	0.108
Mandibular prognathism (mm)	−1.9	7,6	−3.5	6,9	0.018*
Vertical height ratio	1,1	0,1	1	0,1	0.181
Lower face-throat angle (°)	105,1	8,1	106,2	7,9	0.724
Lower vertical height-depth ratio	1,6	1,4	1,4	0,4	0.579
Lip position					
Nasolabial angle (°)	105,7	9,5	108,4	7,4	0.027*
Upper lip protrusion (mm)	3,9	1,7	2,8	2,1	0.041*
Lower lip protrusion (mm)	2,4	1,9	2,1	1,6	0.764
Mentolabial sulcus (mm)	−5.4	1,6	−5.9	1,6	0.034
Vertical lip-chin ratio	0,47	0,1	0,49	0,05	0.527
Maxillary incisor exposure (mm)	2,7	1,8	3,2	1,9	0.216
Interlabial gap (mm)	1,1	1,4	1,1	1,7	0.869

*P* ≥ 0.05–nonsignificant (NS), *P ≤ 0.05, **P ≤ 0.01, ***P ≤ 0.001.

**Table 3:** Soft tissue cephalometric values of different ethnic groups using Legan and Burstone analysis.

Variable	Chinese	Japanese	North Indians	Saudis	Yemeni	Caucasians
Facial convexity angle (°)	10.5 ± 3.5	10.1 ± 5.7	13.34 ± 4.8	15.16 ± 4.64	16.9 ± 5.2	12 ± 4
Maxillary prognathism (mm)	2.5 ± 3	2.3 ± 4.6	5.83 ± 4.3	6.47 ± 4.27	6.9 ± 4.1	6 ± 3
Mandibular prognathism (mm)	N.A.	−5.7 ± 8.3	−1.31 ± 6.4	−1.37 ± 7.19	−4.9 ± 6.7	0 ± 4
Vertical height ratio	1.0 ± 0.1	0.9 ± 0.1	1.03 ± 0.1	1.00 ± 0.09	1.0 ± 0.1	1
Lower face-throat angle (°)	96 ± 4	98.1 ± 9.5	111.57 ± 8.1	102.60 ± 8.24	107.6 ± 7.9	100 ± 7
Lower vertical height-depth ratio	1.1 ± 0.2	1.3 ± 0.2	1.22 ± 0.2	1.14 ± 0.20	1.4 ± 0.2	1,2
Nasolabialangle (°)	95 ± 3	102.3 ± 11.6	95.79 ± 11.4	106.02 ± 11.01	106.4 ± 9.7	102 ± 8
Upper lip protrusion (mm)	7.0 ± 1.5	5.8 ± 2.1	4.72 ± 1.7	3.84 ± 1.56	2.6 ± 1.2	3 ± 1
Lower lip protrusion (mm)	N.A.	5.0 ± 2.5	2.83 ± 1.6	3.26 ± 2.07	2.2 ± 2.2	2 ± 1
Mentolabial sulcus (mm)	3.5 ± 2	4.3 ± 1.4	5.82 ± 1.2	4.60 ± 1.23	5.0 ± 1.1	4 ± 2
Vertical lip-chin ratio	0.5	0.4 ± 0.1	0.44	0.44 ± 0.05	0.4 ± 0.1	0,5
Maxillary incisor exposure (mm)	1.5 ± 1.5	1.8 ± 1.7	2.35 ± 1.5	3.26 ± 1.96	2.9 ± 1.5	2 ± 2
Interlabial gap (mm)	1.0 ± 1.0	1.9 ± 0.9	0.24 ± 0.7	2.24 ± 0.93	0.6 ± 0.4	2 ± 2

N.A.: not available.
